# Factors associated with recognition and prioritization for falling, and the effect on fall incidence in community dwelling older adults

**DOI:** 10.1186/s12877-015-0165-2

**Published:** 2015-12-17

**Authors:** Sofie Jansen, Jolanda Schoe, Marjon van Rijn, Ameen Abu-Hanna, Eric P. Moll van Charante, Nathalie van der Velde, Sophia E. de Rooij

**Affiliations:** Department of Internal Medicine, section of Geriatric Medicine, Academic Medical Center, Meibergdreef 9, 1105 Amsterdam, AZ The Netherlands; Department of Medical Informatics, Academic Medical Center, Amsterdam, The Netherlands; Department of General Practice, Academic Medical Center, Amsterdam, The Netherlands; Department of Geriatric Medicine, University Medical Center Groningen, Groningen, The Netherlands

**Keywords:** Falls, Community-dwelling older persons, Intervention, Priority, Comprehensive geriatric assessment

## Abstract

**Background:**

Recent trials have shown that multifactorial fall interventions vary in effectiveness, possibly due to lack of adherence to the interventions. The aim of this study was to examine what proportion of older adults recognize their falls risk and prioritize for fall-preventive care, and which factors are associated with this prioritization.

**Methods:**

Observational study within the intervention arm of a cluster randomized controlled trial (RCT) on the effect of preventive interventions for geriatric problems in older community-dwellers at risk of functional decline. Setting: general practices in the Netherlands. Participants were community dwellers (70+) in whom falling was identified as a condition. A comprehensive geriatric assessment (CGA) was performed by a registered community care nurse. Participants were asked which of the identified conditions they recognized and prioritized for in a preventive care plan, and subsequent interventions were started. Multivariable logistic regression was performed to identify which factors were associated with this prioritization. Fall-incidence was measured during one-year follow-up.

**Results:**

The RCT included 6668 participants, 3430 were in the intervention arm. Of those, 1209 were at risk of functional decline, of whom 936 underwent CGA. In 380 participants (41 %), falling was identified as a condition; 62 (16 %) recognized this and 37 (10 %) prioritized for it. Factors associated with prioritization for falls-prevention were: recurrent falls in the past year (OR 2.2 [95 % CI 1.1-4.4]), severe fear-of-falling (OR 2.7 [1.2-6.0]) and use of a walking aid (2.3 [1.1-5.0]). Sixty participants received a preventive intervention for falling; 29 had prioritized for falling. Incidence of falls was higher in the priority group than the non-priority group (67 % vs. 37 % respectively) during first six months of follow-up, but similar between groups after 12 months (40.7 % vs. 44.4 %).

**Conclusions:**

The proportion of community-dwellers at risk of falls that recognizes this risk and prioritizes for preventive care is small. Recurrent falls in the past year, severe fear-of-falling and use of a walking aid were associated with prioritization. Prioritization was associated with a greater fall-risk during first six months, which appeared to level out at one-year follow-up. These results could aid in the identification of community-dwellings likely to benefit from fall-preventive interventions.

**Trial registration:**

NTR2653, 17 December 2010

## Background

For many years now, falls in older persons have been recognized as a major and rising healthcare problem. Falls are known to cause physical injury and to limit social and physical activity, resulting in reduced independency and new fall incidents [1–7]. Injurious falls carry an estimated cost of at least 9000 euro per fall [8], and healthcare costs due to falls are likely to increase with ageing of the population [5, 9, 10]. Several studies have investigated whether preventive interventions could reduce the incidence of (recurrent) falls. To date, several meta-analyses show that multifactorial interventions are most effective in reducing rate of falls [9, 11].

Nevertheless, two recent studies were not able to report a positive effect of multifactorial interventions on fall-incidence [12–14]. In these randomized controlled trials it was suggested that lack of adherence to the intervention was an important explanation for these results.

Several reasons for not adhering to fall-preventive interventions have been recognized, of which many are stigma-driven. Potentially, adherence to fall-preventive interventions is better in those who recognize their fall risk and prioritize for fall-preventive care. If we could identify which older people at risk of falls recognize this risk and wish to undergo preventive treatment for it, this may help to identify community-dwelling older people who are more motivated to undergo a fall-preventive intervention and therefore are more likely to benefit from such an intervention.

In this study, we therefore studied what proportion of older community-dwelling adults at risk of falls preferred to prioritize for treatment and/or preventive care for falls, and what characteristics were associated with this prioritization. Furthermore, we studied fall incidence in those who underwent an intervention for falls, and differences in fall incidence between those who prioritized and those who did not.

## Methods

### Population

This study was part of a cluster randomized controlled trial that investigates whether functional decline in community-dwelling older persons can be delayed or prevented through a nurse-led multifactorial preventive intervention [20]. The study is a multifactorial and multidisciplinary study assessing effectiveness of preventive interventions in older people in the general practice. The 24 practices, located in the north-western region of the Netherlands, were cluster randomized per practice (intervention or control). Approximately 10,000 community-dwelling older persons aged 70 years or over were eligible for inclusion. Exclusion criteria were: terminal illness, dementia, no understanding of Dutch, planning to move or spend a long time abroad or planning to move to a nursing home. Eligible persons received a letter with information from their general practitioner (GP) about the study and invitation to participate. A detailed description on patient recruitment and enrolment has been reported previously [20]. All participants provided signed informed consent prior to taking part in the study. To prevent selection bias, a postponed informed consent procedure was used to blind all participants in both study arms [20]. In the intervention condition, eligible participants were further informed about the procedure of the intervention, but they were not otherwise informed that this was the intervention under study. As explained in the study information, participants in both study groups received written information on the complete study objectives and outcomes after termination of the study.

For inclusion in the present study, participants were eligible if they were at risk of functional decline and thus underwent comprehensive geriatric assessment (CGA. The study was approved by the Medical Ethics Committee of the Academic Medical Centre (protocol ID MEC10/182). All experimental procedures adhered to the Declaration of Helsinki. All participants provided signed informed consent prior to taking part in the study.

### Comprehensive geriatric assessment (CGA)

The Identification of Seniors at Risk Primary Care score (ISAR-PC) was used to identify participants at risk of functional decline [21]. This score was calculated for both study arms. All participants within the intervention arm with a positive ISAR-PC (score ≥2) were invited for CGA and subsequent interventions.

The CGA contained short yes/no questions about participant’s health and functioning and addressed frequently encountered geriatric conditions, including falls. A specially trained registered community care nurse conducted the systematic CGA. During the second home visit, further diagnostic assessments followed for the identified conditions based on standardized protocols. Subsequently, the diagnostic yield of both home visits was discussed with the GP to develop an individually tailored CTP. The CTP contained interventions derived from toolkits for the specific geriatric problems [22]. These toolkits consist of standardized protocols and are based on national GP guidelines [23]. The assembly of the toolkits is described in more detail in study protocol [20]. After this meeting, a third home visit was used to discuss the CTP with the participants and their caregivers. Potential discrepancies between the priorities of the patients, RNs and GPs was addressed to find a consensus on the CTP.

### Identification of fall risk and subsequent interventions

During the CGA, participants were asked how often they had fallen in the past year. Recurrent falls were defined as two or more falls in the past year. Fear-of-falling was indicated on a visual analogue scale (1–10) by the participant; a score of one or more on this scale indicated fear-of-falling [3]. Severe fear-of-falling was identified as a score of five or more on this scale. Falling was identified as a condition in the CGA if the participant had fallen one or more times in the past twelve months and/or if they expressed fear-of-falling.

After the CGA, participants were asked whether they recognized the identified geriatric condition(s). Subsequently, they were asked whether they wanted any help with or treatment for them, and in case of multiple issues, with which set of problems they would prefer to start. This was categorized as recognition and prioritization. Preventive interventions for falling were derived from the toolkit *fall and fracture risk* [24]. This toolkit covers the following risk factors and points to consider: impaired mobility, use of psychotropic drugs, polypharmacy, impaired ADL, reduced physical activity, impaired vision, urine incontinence, depressive symptoms, cognitive dysfunction, female sex, age, cardiovascular factors, syncope, osteoporosis and (home-) safety. Depending on the risk factor, the toolkit recommends an intervention or refers to another specific toolkit.

The intervention took place during a twelve-month period. During the study period, the nurse paid one or more (up to eight) home visits to the participants to motivate them and help implement the interventions. These visits were spread over the follow-up period of 12 months; the total number of visits was aimed at three to eight visits per participant. The GP, the nurse, a specialized therapist and/or the participant carried out the interventions. Participants in both arms of the study were asked to fill in a self-completion questionnaire (SCQ) at baseline and after six and twelve months. These questionnaires contained questions about health-related problems, such as (instrumental) activities of daily life (ADL/iADL), quality of life, comorbidity, medication and cognitive function, including several risk factors for falling.

Fall incidence during follow-up was measured at six and twelve months through the SCQ, by asking participants how often they had fallen in the past six months.

### Outcome measures

*Primary outcome measure* was the identification of variables that were associated with recognition and prioritization of fall risk. This was investigated within all participants in whom falling was identified as a condition in the CGA.

*Secondary outcome measure* was the difference in incidence of fall-incidents during follow-up between those who prioritized falling and those who did not, in participants who received an intervention for falling.

### Covariates of interest

At baseline, a multitude of variables was gathered through the SCQ.

*Socio*-*demographic variables* included age, sex, country of origin, living situation and marital status. Socio economic status (SES) was derived from the postal area of respondents.

*Functional variables* included the use of household or daily care assistance, involuntary urine loss, use of incontinence material, impaired hearing, impaired vision and use of a walking aid.

*Comorbid conditions* included diabetes, cerebrovascular accident or transient ischemic attack (CVA/TIA), heart failure, myocardial infarction or angina, malignancy, asthma or chronic bronchitis, osteoarthritis, osteoporosis, hip-fracture, other fractures, dizziness and prostate problems.

*Mental and social health variables* included self-reported health, quality of life (EQ-5D utility), subjective memory loss, depression, anxiety or panic disorder, and dementia.

*Medication use* included polypharmacy (≥3 medications) and number of all medications used in case of polypharmacy.

### Statistical analyses

Conventional statistics were used to compare differences between groups. Categorical variables were compared using Chi squared tests. Continuous variables were compared using independent samples T-tests and Mann–Whitney-U tests in case of non-normal distribution of variables.

All covariates that were univariately associated with recognition and prioritization with a p-value of <0.25 were tested for an association after adjustment for potential confounders [25]. Variables were entered into separate multivariable models with age, sex, primary education, socioeconomic status, and covariates that acted as potential confounders. Covariates were tested for potential confounding by adding them to the model with the variable that was associated with the dependent variable. If the covariate changed the odds ratio (OR) by 10 % or more was it was considered a potential confounder, except if this particular covariate was considered a mediator rather than a confounder.

The following factors were tested for potential confounding: use of a mobility aid, severe fear-of-falling, recurrent falls in the past year, marital status, living situation, visual problems, hearing problems, daily care assistance, depression, involuntary loss of urine, use of incontinence material, medial history of CVA/TIA, dementia and anxiety or panic disorder and quality of life.

To measure differences in fall-incidence during follow-up between groups, we also adjusted for baseline differences. Multivariable logistic regression analyses were used for dichotomous covariates, and Poisson regression analyses for continuous variables because of non-normal distribution of fall-incidence during follow-up. Statistical analyses were performed using SPSS (version 20). A *p*-value of <0.05 was considered statistically significant.

## Results

**Fig. 1:**
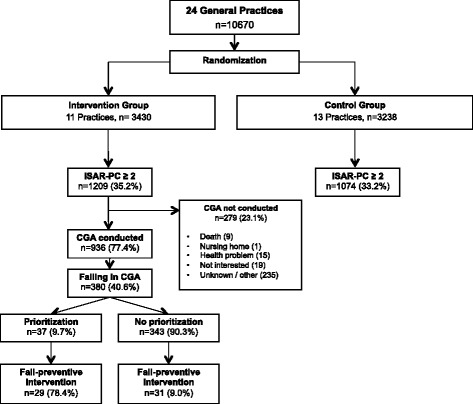
Flowchart of patient inclusion

The RCT included 6668 participants of whom 3430 were in the intervention arm (Fig. 1). Of those, 1209 were at risk of functional decline, of whom 936 underwent CGA. In 380 participants (41 % of those at risk of functional decline), falling was identified as a condition; 16 % (*n* = 62) patients recognized this risk and 10 % (*n* = 37) of participants prioritized for treatment and/or prevention for falling. Of these participants, 78 % (*n* = 29) received an intervention for falling. Due to the postponed informed consent procedure there was a loss of participants in the ISAR-PC positive group of the intervention arm, as they found the full extent of the study too time consuming or overwhelming.

### Prioritization of falling

Table 1 shows the baseline characteristics of participants for whom falling was identified as a condition in the CGA, comparing those who prioritized falling to those who did not. Mean age of the priority group was 81.7 (±6.0) years and 81.9 (±6.2) years in the non-priority group. Both groups consisted mainly of women and the majority of participants was born in the Netherlands. Those in the priority group reported more falls in the past year, and more often reported severe fear-of-falling.

**Table 1:** Baseline characteristics of participants for whom falling was identified as a condition, comparing those who prioritized falling to those who did not

	Priority for falling (*n* = 37)	No priority for falling (*n* = 343)	*p*
*Sociodemographic variables*			
Age	81.7 (±6.0)	81.9 (±6.2)	0.860
Sex, female	29 (78.4 %)	239 (69.7 %)	0.270
Born in The Netherlands	36 (97.3 %)	327 (96.2 %)	0.732
Socioeconomic status			
-Low (≤1SD)	0 (0.0 %)	17 (5.0 %)	0.165
-Intermediate	33 (89.2 %)	258 (75.4 %)	**0.060**
-High (≥1SD)	4 (10.8 %)	67 (19.6 %)	0.194
Highest level of education			
-Primary school or less	8 (21.6 %)	109 (32.2 %)	**0.189**
-Secondary education	23 (62.2 %)	183 (54.0 %)	0.342
-Tertiary education	6 (16.2 %)	47 (13.9 %)	0.696
Living situation			
-Alone	20 (54.1 %)	160 (47.1 %)	0.419
-With partner	14 (37.8 %)	144 (42.4 %)	0.597
-Nursing home	3 (8.1 %)	36 (10.6 %)	0.638
Marital status			
-Married	16 (43.2 %)	141 (41.3 %)	0.824
-Divorced	4 (10.8 %)	14 (4.1 %)	**0.069**
-Widowed	16 (43.2 %)	157 (46.0 %)	0.746
-Unmarried	1 (2.7 %)	29 (8.5 %)	**0.215**
*Falls*			
Falls in the past year (≥1)	21 (58.3 %)	195 (57.5 %)	0.925
Recurrent falls in the past year (≥2)	16 (45.7 %)	92 (28.1 %)	**0.031**
Number of falls in the past year	1.0 [0.0; 3.0]	1.0 [0.0; 2.0]	**0.160**
Severe fear-of-falling	20 (64.5 %)	131 (39.9 %)	**0.008**
*Functioning*			
Impaired hearing	21 (56.8 %)	164 (48.2 %)	0.325
Impaired vision	18 (48.6 %)	145 (42.8 %)	0.493
Use of walking-aid	25 (67.6 %)	172 (51.0 %)	**0.056**
Household assistance	24 (64.9 %)	237 (70.1 %)	0.510
Home care	5 (13.5 %)	60 (17.6 %)	0.527
*Mental health and Quality of life*			
Depression	4 (10.8 %)	34 (10.0 %)	0.876
Anxiety-/panic disorder	3 (8.1 %)	25 (7.4 %)	0.868
Dementia	1 (2.7 %)	6 (1.8 %)	0.688
Subjective memory loss	9 (24.3 %)	107 (31.8 %)	0.348
Self-reported health, less than good	19 (51.4 %)	193 (56.6 %)	0.541
EQ-5D Utility	0.8 (±0.1)	0.7 (±0.2)	**0.207**
Quality of life, less than good	11 (29.7 %)	92 (28.0 %)	0.821
Quality of life (scale 1–10)	7.2 (±1.1)	7.2 (±1.1)	0.836
*Comorbidity*			
Polypharmacy (3 or more medications)	26 (70.3 %)	241 (71.1 %)	0.917
Nr. of drugs (if polypharmacy reported)	6.1 (±3.1)	5.6 (±2.8)	0.410
Diabetes	6 (16.2 %)	60 (17.6 %)	0.828
CVA/TIA*	6 (16.2 %)	25 (7.4 %)	**0.062**
Heart failure	4 (10.8 %)	62 (18.2 %)	0.259
Myocardial infarction/angina	4 (10.8 %)	23 (6.8 %)	0.365
Asthma/chronical bronchitis	6 (16.2 %)	55 (16.2 %)	0.995
Involuntary urine loss	11 (29.7 %)	108 (31.8 %)	0.800
Use of incontinence material	20 (54.1 %)	163 (49.5 %)	0.603
Osteoarthritis	22 (59.5 %)	200 (59.0 %)	0.957
Osteoporosis	11 (29.7 %)	85 (25.0 %)	0.531
Hip-fracture	2 (5.4 %)	18 (5.3 %)	0.977
Other fractures	3 (8.1 %)	34 (10.0 %)	0.713
Dizziness	15 (40.5 %)	115 (33.8 %)	0.414
Prostate problems	2 (5.4 %)	32 (9.4 %)	0.417

Notes: data are mean (±SD), *n* (%) or median [IQR]. CVA/TIA = Cerebrovascular accident/transient ischemic attack. Bold values indicate significant results with *p* < 0.250

Table 2 shows the results of the multivariable regression analyses, showing variables that were univariately associated with prioritization (*p* < 0.250). These variables were subsequently adjusted for age, sex, primary education, socioeconomic status and covariates that acted as potential confounders. After adjustment for potential confounders, the following variables were independently associated with prioritization: recurrent falls in the past year (OR 2.2 [95 % CI 1.1–4.4]), fear-of-falling (OR 2.7 [1.2-6.0]) and use of a walking aid (2.3 [1.1–5.0]).

**Table 2:** Factors associated with prioritization for falling in participants for whom falling was identified as a condition (priority group *n* = 37, non-priority group *n* = 343)

Covariates	Unadjusted			Adjusted		
	OR	95 % CI	*P*	OR	95 % CI	*P*
Highest level of education is primary or less	1.7	(0.8–3.9)	0.193	N/S		
Socioeconomic status^c^	1.1	(005–2.2)	0.816	N/S		
EQ-5D utility	3.5	(0.5–25.1)	0.209	N/S		
Divorced	2.5	(0.7–8.6)	0.140	N/S		
CVA/TIA	2.4	(0.9–6.4)	0.070	2.6	(0.97–6.9)	0.057
Use of walking-aid^a^	2.0	(0.97–4.1)	0.060	2.3	(1.1–5.0)	0.035
Severe fear-of-falling^b^	2.7	(1.3–5.9)	0.010	2.7	(1.2–6.0)	0.019
Recurrent falls in the past year (≥2)	2.2	(1.1–4.4)	0.034	2.2	(1.1–4.4)	0.031

*CVA*/*TIA* cerebrovascular accident/transient ischemic attack, *N*/*S* non-significant

Final model adjusted for age, sex, primary education and socioeconomic status

^a^Final model additionally adjusted for anxiety or panic disorder

^b^Final model additionally adjusted for EQ5D utility score and use of a walking aid

^c^entered as a continuous variable

### Fall incidence

Table 3 shows the baseline characteristics of all participants who received an intervention for falling (priority group *n* = 29, non-priority group *n* = 31). Mean age was 81.7 (±5.8) years in the priority group and 80.9 (±6.4) years in the non-priority group. At baseline, number of falls in the past year was significantly higher in the priority group. Other covariates were equal in both groups.

**Table 3:** Baseline characteristics of participants who received an intervention for falling, comparing those who prioritized falling to those who did not

	Priority for falling (*n* = 29)	No priority for falling (*n* = 31)	*p*
Sociodemographic variables			
Age	81.7 (±5.8)	80.9 (±6.4)	0.624
Sex, female	21 (72.4 %)	20 (64.5 %)	0.511
Born in the Netherlands	28 (96.6 %)	29 (93.5 %)	0.594
Socioeconomic status			
-Low (≤1SD)	0 (0.0 %)	1 (3.2 %)	0.329
-Intermediate	26 (89.7 %)	19 (61.3 %)	0.011
-High (≥1SD)	3 (10.3 %)	11 (35.5 %)	0.012
Highest level of education			
-Primary school or less	6 (20.7 %)	5 (16.1 %)	0.648
-Secondary school	18 (62.1 %)	20 (64.5 %)	0.844
-Tertiary school	5 (17.2 %)	6 (19.4 %)	0.833
Living situation			
-Alone	17 (58.6 %)	18 (58.1 %)	0.965
-With partner	10 (34.5 %)	12 (38.7 %)	0.734
-Nursing home	2 (6.9 %)	1 (3.2 %)	0.514
Marital status			
-Married	12 (41.4 %)	10 (32.3 %)	0.464
-Divorced	2 (6.9 %)	4 (12.9 %)	0.438
-Widowed	14 (48.3 %)	14 (45.2 %)	0.809
-Unmarried	1 (3.4 %)	3 (9.7 %)	0.334
Falling and functioning			
Falls in the past year (≥1)	18 (62.1 %)	22 (71.0 %)	0.465
Recurrent falls in the past year (≥2)	14 (50.0 %)	8 (26.7 %)	0.067
Number of falls in the past year	3.0 [2.0; 4.0]	1.0 [1.0; 2.0]	0.004
Severe fear-of-falling	17 (68.0 %)	15 (50.0 %)	0.178
Use of a walking aid	17 (58.6 %)	18 (58.1 %)	0.965
Mental health and quality of life			
Self-reported health, less than good	14 (48.3 %)	18 (58.1 %)	0.448
Quality of life, less than good	9 (31.0 %)	9 (29.0 %)	0.866
EQ-5D Utility	0.8 (±0.1)	0.7 (±0.3)	0.260

Notes: data are mean (±SD), *n* (%) or median [IQR]

Table 4 shows fall incidence during follow-up in participants that received a preventive fall intervention, comparing the priority-to the non-priority group. During one-year follow-up, proportion of falls and recurrent falls was higher in the priority group than the non-priority group (falls: 78 % vs. 58 %, recurrent falls: 56 % vs. 35 %, number of falls: 2.0 [IQR 0.5; 3.5] vs. 1.0 [0.0; 2.0] respectively), but these differences were not statistically significant. During first half (months 0–6) however, there was a significantly higher incidence of falls and recurrent falls in the priority group. During second half of follow-up (months 7–12), fall incidence was equal in both groups.

**Table 4:** Incidence of falls during follow up in participants who received a preventive intervention for falling, comparing those who prioritized falling to those who did not

	Priority for falling (*n* = 29)	No priority for falling (*n* = 31)	*p*
Total follow-up (0–12 months)			
Falls (≥1)^c^	21 (77.8 %)	15 (57.7 %)	0.117
Recurrent falls (≥2)^c^	15 (55.6 %)	9 (34.6 %)	0.126
Number of falls^d^	2.0 [0.5; 3.5]	1.0 [0.0; 2.0]	0.128
First half (0–6 months)			
Falls (≥1 event)^a^	18 (66.7 %)	10 (37.0 %)	0.029
Recurrent falls (≥2 events) ^a^	14 (51.9 %)	6 (22.2 %)	0.024
Number of falls^a^	2.0 [0.0; 3.0]	0.0 [0.0; 1.0]	0.007
Second half (7–12 months)			
Falls^a^	11 (40.7 %)	12 (44.4 %)	0.783
Recurrent falls^b^	6 (23.1 %)	5 (18.5 %)	0.682
Number of falls^b^	0.0 [0.0; 1.25]	0.0 [0.0; 1.0]	0.802

^a^data missing for 2 participants in the priority group and 4 in the non-priority group. ^b^data missing for 3 partipants in the priority group and 4 in the non-priority group. ^c^data missing for 2 participants in the priority group and 5 in the non-priority group. ^d^data missing for 4 participants in the priority group and 5 participants in the non-priority group

Data are n (%) or median [IQR]

To adjust for baseline differences between groups, the association between fall incidence and priority for falling was calculated through multivariable logistic regression. Unadjusted, fall incidents and recurrent fall incidents within six months of follow-up were significantly associated with priority for falls (OR 3.4 [1.1-10.4] and 3.8 [1.2-12.3] respectively), but after adjustment for age, sex, SES and number of falls at baseline, these associations were no longer significant. During second half of follow-up, falls and recurrent falls were not associated with priority for falling, and neither during entire follow-up.

Number of falls during complete follow-up was higher in the priority group, but not significantly different (Incidence rate ratio [IRR] 1.9 (95%CI 0.96-3.8), *p* = 0.063). Adjustment for age, sex, SES, number of falls at baseline and severe fear-of-falling at baseline reduced the IRR; results were not significant. Number of falls during first half of follow-up was higher in the priority group (IRR 3.1 [1.5-6.1], *p* = 0.001). Adjustment for age, sex, SES, number of falls at baseline and severe fear-of-falling led to a reduction in the IRR, but did not alter significance of this result (IRR 2.2 [1.1-4.3], *p* = 0.023). Unadjusted and adjusted for baseline differences, number of falls during second half of follow-up was not significantly different between groups.

## Discussion

In this study on preventive interventions to reduce functional decline in community-dwelling older people, almost half of participants who underwent a nurse-led comprehensive geriatric assessment were identified to be at risk of falls. Only sixteen per cent recognized this fall risk, and ten per cent of these older adults prioritized to undergo preventive care or treatment for falls. Use of a walking aid, severe fear-of-falling and a history of recurrent falls were positively associated with prioritization for falling. Prioritization was associated with a greater fall-risk during first six months, but after twelve months there was no difference in fall incidence between those who did and did not actively prioritize for falling.

Use of a walking aid, severe fear-of-falling and a history of recurrent falls were positively associated with recognition and prioritization for falling. All are known risk factors for falling [26], and these results might indicate that those with multiple fall incidents who develop fear-of-falling may perceive themselves at higher risk of falls than those with a history of a single fall incident and/or no fear of falls. Hughes et al. [27] found that over 60 % of community dwelling older adults perceive their fall risk as low, and they reported that absent history of falls and a better self-reported health were associated with a lower self-perceived risk of falls. Furthermore, those who reported a low priority for them were also more likely to report a self-perceived lower risk of falling. This is in concordance with our results. Use of a mobility aid was also associated with prioritization. Previous studies have shown that older adults are often reluctant to use mobility aids and frequently mentioned barriers include denial of need, fear of dependence and stigma, and embarrassment [17]. That use of a mobility aid was positively associated with prioritization may indicate that this group has already overcome the barriers to use a mobility aid, reducing the threshold to engage in a preventive intervention. Furthermore, it is likely that participants using a mobility aid have more mobility problems than those who do not use mobility aids. Perhaps this leads to greater awareness of their fall risk and more motivation to undergo a preventive intervention. As this study did not measure adherence, we were unable to study the effect of prioritization on actual adherence to the intervention.

To date, only a few studies regarding participants’ adherence have been performed, most of which have reported on subjective measures based on qualitative research [15–19, 28].

Most frequently mentioned reasons for not adhering to the interventions were costs [15], transportation and family burden [16] and not wanting to appear as old and frail, with or without mobility aids [17–19]. Sjösten et al. studied subjective and objective predictors of adherence in a multifactorial falls prevention trial [29]. Contrary to our results, they found that participants with a lower self-perceived risk of falling at home were more adherent to the prevention programme. They also found better adherence among women and participants with good cognitive and physical functioning.

Incidence of falls was higher in the priority group during first six months of follow-up but levelled with the non-priority group after twelve months. It is likely that the higher incidence and number of falls in the priority group during the first half of follow-up is due to their higher baseline risk of falling.

Hypothetically, the levelling of the difference in fall incidents during second half of the intervention could be due to greater adherence to the intervention in the priority group. As interventions in the current study were on-going during follow up, they were possibly not yet effective in the first half of follow-up. Potentially, participants who prioritized for falling were more adherent to the intervention, resulting in a better effect of the intervention on fall incidence in the long term (second half of first-year follow-up). This is however speculative, and the small sample size and large baseline differences between the groups hinder us to draw robust conclusions from these findings.

Furthermore, not all fall risk-increasing factors are permanent, such as reduced mobility after illness, which may explain why the differences in fall incidence levelled during the course of the intervention.

Our study has some limitations. Despite the fact that the trial included a large sample size, the amount of participants that recognized and prioritized for falls as a problem was small, resulting in a small sample available for the current analyses. Nevertheless, there was a good distribution of participants with and without priority for falling in the fall-intervention group. Furthermore, participants who recognized and prioritized falling may have reported on falls more accurately during follow-up than those who did not, leading to an under-report of falls in the non-priority group. However, both groups in the sample in which fall incidence was measured underwent an intervention for falls, and it is known that recall of falls is better in those undergoing an intervention [30]. Furthermore, information on falls was collected retrospectively at six-month intervals, which is likely to have resulted in underreport [31]. Also, our study was part of a multifactorial preventive intervention for different geriatric conditions. Multiple problems with subsequent interventions could emerge from the CGA, resulting in a multitude of interventions and a difference in ranking of priority for these problems. Fall-preventive interventions could therefore have received less attention, resulting in less effectiveness. Also, we cannot exclude that interventions aimed at other geriatric conditions had a preventive effect on fall incidents as well, for instance medication review because of polypharmacy, thus attenuating the results. Furthermore, since the CGA and subsequent intervention was carried out by thirteen community care nurses, potential inter-individual differences in recognition of fall risk and subsequent intervention techniques between study nurses cannot be excluded. All study nurses however attended joint training sessions, minimalizing these differences.

A higher self-perceived risk of falling could lead to increased priority for falls prevention, resulting in better adherence to a preventive intervention. As adherence is a very important determinant of success in fall-prevention programmes, programmes should strive to optimise this factor. Studies investigating the effectiveness of fall prevention programmes should therefore add adherence to their measures, as only a few studies have researched adherence and associated factors to date. As the follow-up of this study is ongoing, we will be able to assess the effect of priority for fall-incidence in future waves of the study.

## Conclusion

In summary, only ten per cent of community dwelling older persons in a primary care setting prioritized for falls in a primary care based preventive intervention. Recognition and prioritization for falling was associated with history of recurrent falls, severe fear-of-falling and use of a walking aid. Those who recognized their fall risk and prioritized for falls prevention fell more often during first six months of follow-up. The results of this study may be helpful in identifying which community dwelling older adults are most likely to benefit from fall-preventive interventions. Potentially, this could make fall-preventive interventions more cost-effective.

## Abbreviations

ADL/IADL: (Instrumental) activities of daily living
CGA: Comprehensive geriatric assessment
CTP: Care and treatment plan
CVA: Cerebrovascular accident
EQ-5D: Quality of life. range: 0–1; 0 indicates very poor quality of life 1 indicates a very good quality of life
GP: General practitioner
ISAR-PC: Identification of seniors at risk primary care score
SCQ: Self-completion questionnaire addressing health and functioning
SPSS: statistical package for the social sciences computer software for statistical analyses
TIA: transient ischemic attack
